# Outcomes of Anorexia Nervosa in a Male Patient Treated With Paroxetine: A Case Report

**DOI:** 10.7759/cureus.58765

**Published:** 2024-04-22

**Authors:** Mohammed Alkhamis, Waad D Alotaibi, Ghaiah J Alharbi, Anwar M Alsaeed, Fatimah A Almuhaysin

**Affiliations:** 1 Psychiatry, King Fahad Hospital, Al-Hofuf, SAU; 2 Medicine, King Faisal University, Al-Hofuf, SAU

**Keywords:** eating behaviour, eating behaviors, paroxetine, anorexia nervosa, eating disorders

## Abstract

Eating disorders (EDs) are among the most dangerous mental illnesses, that are characterized by high mortality rates, multisystem comorbidity, and an often chronic and relapsing disease course. EDs occur most commonly in the female gender, with a ratio of 10 females to 1 male for anorexia nervosa (AN). We present the case of a 15-year-old Saudi boy who presented with weight loss (BMI 11.6 kg/m^2^) and began to have symptoms of obsessive-compulsive disorder (OCD) in prayer and ablution. His first treatment plan was psychoeducation. He then developed a fear of gaining weight and began to count calories; he was diagnosed with AN and started on olanzapine 2.5 mg. The patient had a history of multiple admissions due to electrolyte imbalance, hypokalemia, hypoglycemia, and anal fissure due to constipation, and was prescribed olanzapine 5 mg, fluoxetine 20. His last admission was the worst, as he became semi-comatose with a Glasgow Coma Scale (GCS) of 13, was diffused and disoriented to time and person, unable to walk or sit, and was uncooperative in answering questions. During admission, we changed the fluoxetine to paroxetine 25 mg and increased the olanzapine to 10 mg, and the patient showed a huge improvement physically and mentally. This case emphasizes the significance of including paroxetine in the treatment of diagnoses for AN to prevent unnecessary wasting of time and effort.

## Introduction

Eating disorders (EDs) are among the most dangerous mental illnesses. They are characterized by high mortality rates, multisystem comorbidity, and an often chronic and relapsing disease course [1], and occur more in females rather than males, with a 10:1 female-to-male ratio for anorexia nervosa (AN) [2]. There are limited studies on AN in males due to a very small percentage of cases. This has led to complications for males, such as delayed treatment and medical complications, including loss of growth potential and cardiovascular sequelae [3].

This case report aims to shed light on a less commonly encountered but important condition in the context of AN for the male gender. During the 1990s, systematic research sought to examine the prevalence and correlates of male AN cases in specialist ED clinics, noting that males represented approximately 5-10% of cases [1]. We describe a rare case in which a male presented with AN, underscoring the importance of the awareness and consideration of this condition when evaluating patients with an ED.

## Case presentation

The paper presents the case of a 15-year-old Saudi male, who was studying in secondary school, single, had a twin, and lived with his mother and siblings; his father died 11 years ago due to a chronic illness. He was medically free and presented in the clinic with anorexia and decreased oral intake. He was also complaining of heartburn, for which he was given proton pump inhibitors with no improvement. He had weight loss, with a weight of 30.5 kg and a height of 162 cm (BMI 11.6 kg/m^2^). His symptoms did not improve, so he underwent an upper and lower gastrointestinal endoscopy, which was normal. The patient subsequently began to show symptoms of obsessive-compulsive disorder (OCD) in prayer and ablution; he was seen by a psychiatrist for the first time and his weight had reduced slightly to 29.3 kg (BMI 11.2 kg/m^2^). The initial treatment plan was psychoeducation. By the second visit for follow-up, his OCD symptoms had improved, but his family had noticed that he was hiding food, secretly exercising, had a fear of gaining weight, and was counting calories. His weight at that time was lower at 25.1 kg (BMI 9.6 kg/m^2^), so he was diagnosed with AN and started on olanzapine 2.5 mg and given a dietitian referral.

During the following year, he had multiple clinic visits and four hospital admissions. He was mainly admitted for electrolyte imbalance, hypokalemia, hypoglycemia, and anal fissures due to constipation. He was also given Ensure liquid milk as a nutritional supplement, olanzapine 5 mg, fluoxetine 20 mg, and mirtazapine, which he took for a while and then discontinued because of the side effects. During this period, he refused to measure his weight, was eating very little and only under the pressure of his family, and was having feelings of guilt after eating. He liked having a thin body but was aware that he had reached an unhealthy weight. The patient’s last hospital admission was the worst, as he became semi-comatose with a Glasgow Coma Scale (GCS) of 13, was diffused and disoriented to time and person, was unable to walk or sit, and was uncooperative when asked questions. During admission, he was agitated and screamed when he saw the intravenous line fluid, as he thought a lot of calories would enter his body, so we decided to hide the bottle of fluid. We also changed his fluoxetine 40 mg to paroxetine 25 mg and increased the olanzapine to 10 mg.

After the patient was stabilized medically, he was discharged. He was later seen in an outpatient clinic; he had much improved and had started to walk and talk, and he was more cooperative than before. His cognitive functions were wholly intact, but when we talked about food, the patient delayed his response or ignored our questions. His weight had increased to 33 kg, and while his family reported he was still stubborn and resisted eating, he was more flexible than before. With the next follow-up, the paroxetine dose was increased to 62.5 mg and the olanzapine to 15 mg, and there was a slow improvement regarding concentration, school achievement, and less resistance to eating. We involved psychologists for cognitive behavior therapy (CBT), but there was little improvement or even compliance with following the program. There was no history of substance use or mood issues for more than two weeks that were not related to food, no OCD symptoms, and no history of hallucinations or delusions, but the patient has a history of disorganized behavior, such as hiding between cars or talking to himself, but this had stopped one year ago. There was no need for admission since paroxetine was included in his management plan and the patient was still under follow-up.

## Discussion

AN is thought to be a female disorder [4]. Due to the reliance on amenorrhea as a diagnostic criterion in DSM-IV and the widely held belief that AN is a condition commonly seen in adolescent females, it is uncommon to see clinical presentations of AN in male patients, and its incidence is underestimated [4]. Biases about EDs that are based on gender may prevent diagnosis and treatment initiation in males with AN until severe medical complications occur [1].

The number of males suffering from EDs has risen by 28.9% [3]. In DSM-V, the amenorrhea criterion has been removed and the BMI-based severity rating has been introduced, which represents a huge improvement in the diagnostic definition for males [3].

Males and females with AN present with many similarities. However, male patients are more likely to have a history of premorbid obesity, psychiatric comorbidities, and later age of onset, but they are less likely to have a history of suicide attempts, and typically have shorter durations of hospitalization and illness [1-3]. Moreover, males tend to exhibit some clinical traits more frequently than women. The relationship profile in males also tends to differ relative to their rumination on their condition, and males with AN generally report having less friendly and romantic relationships [2].

ED behaviors such as extreme dieting, laxative abuse, self-induced vomiting, and prolonged fasting are reported to be increasing at a faster rate in males compared to females [5,6]. Over-exercising is also seen more in adolescent males with AN, which is considered to be an attempt to maintain control over pubertal changes [7].

The medical management of AN on a general ward is challenging. However, it is important to identify young people who are at risk of medical complications, so that early intervention can be instigated [8]. Involving ED specialists who sometimes have psychiatry or adolescent medicine expertise - is beneficial, but access to such specialists might be limited in some places. A dietician can assist in choosing calorie-dense, nutrient-rich foods. It is imperative that schools are involved in the identification and treatment of youth with AN [9]. Furthermore, each patient should receive individualized care based on the severity of their symptoms, the progression of the disease, mental comorbidities, the availability of family and social support, and the accessibility of specialized treatment programs in the area. Among the factors that warrant hospitalization are significant electrolyte imbalances, arrhythmias or severe bradycardia, rapid and sustained weight loss despite outpatient care, and major concomitant medical or psychiatric disorders, including suicidal ideation [10,11].

Regarding the outcomes for AN patients, there is no scientific data to recommend the use of one therapeutic setting over another [11]. Significant progress in the treatment of AN, especially in adolescents, points to the efficacy of specific family-based approaches. Although no one treatment has been proven to be superior, it is possible for adults with AN to recover or at least significantly improve [12].

The most successful treatment plan may involve a combination of AN-specific psychotherapy and re-nourishment [13]. Anorexic patients may consume as little as 500 calories per day, while, on average, female adolescents who are sedentary should consume 1,800 calories compared to 2,200 for males [14].

The most important element of treating an ED is psychotherapy. One of the more beneficial treatment modalities for teenagers with AN is family-based treatment [15-17]. Fluoxetine (an SSRI) does not appear to add significant benefit to the inpatient treatment for AN, but it might decrease the symptoms of despair and thoughts of suicide in anorexic patients [18].

## Conclusions

AN is a potentially life-threatening ED. Patients with AN have excessive weight loss and patients should only be diagnosed with AN after all necessary investigations have excluded any medical cause. Treating AN requires a combination of psychotherapy, medication, nutritional counseling, and medical care. AN might lead to medical, social, and psychological complications; therefore, family members and physicians should be aware of the potential for complications.
